# Supplementary material for: Metabolic interdependence and rewiring in radiolaria-microalgae photosymbioses
Source: ISME J. 2025 Mar 9;19(1):wraf047. doi: 10.1093/ismejo/wraf047 (PMC11965087; doi:10.1093/ismejo/wraf047)
Supplement: Supplementary_information_wraf047 [file supplementary_information_wraf047.pdf]

Supplementary Information:

Metabolic Interdependence and Rewiring in Radiolaria-Microalgae Photosymbioses

Vera Nikitashina[1], Benjamin Bartels[2], Joost Samir Mansour[3], Charlotte LeKieffre[4], Johan Decelle[4], Christian Hertweck[2,5,6], Fabrice Not[3], and Georg Pohnert[1,6*]

[1]Institute for Inorganic and Analytical Chemistry, Friedrich Schiller University Jena – Jena, Germany

[2]Department of Biomolecular Chemistry, Leibniz Institute for Natural Product Research and Infection Biology (Leibniz-HKI) – Jena, Germany.

[3]Adaptation and Diversity in Marine Environment (AD2M) Laboratory, Ecology of Marine Plankton Team, Sorbonne Université, CNRS, Station Biologique de Roscoff, UMR7144 - AD2M – Roscoff, France

[4]Laboratoire Physiologie Cellulaire et Végétale, CNRS, CEA, INRAe, IRIG-LPCV – Grenoble, France

[5]Faculty of Biological Sciences, Friedrich Schiller University Jena – Jena, Germany

[6]Cluster of Excellence Balance of the Microverse, Friedrich Schiller University Jena, Jena, Germany

*contact Georg Pohnert: Georg.Pohnert@uni-jena.de

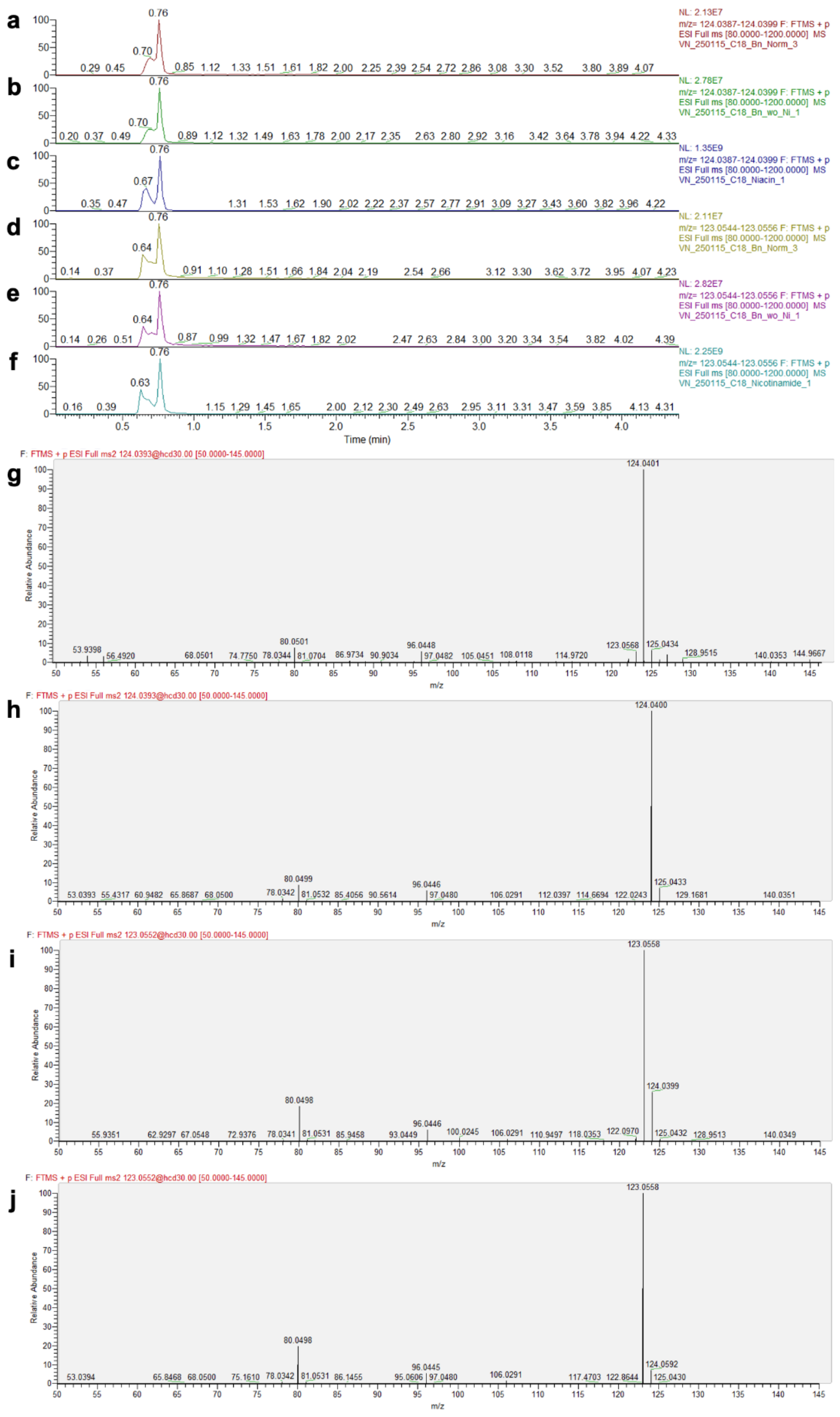

Appendix S1: Extracted ion chromatograms for niacin – *m/z* 124.0394 ± 5ppm (**a-c**) and nicotinamide – *m/z* 123.0553 ± 5ppm (**d-f**), for *Brandtodinium nutricula* cultivated with niacin (**a**, **d**) and without niacin (**b**, **e**), and niacin (**c**) nicotinamide (**f**) standard; MS2 spectra of niacin (**g**, **h**) and nicotinamide (**i**, **j**) for *B. nutricula* extracts (**g**, **i**) and pure niacin (**h**) and nicotinamide (**j**).

Appendix S2: MALDI-2-MSI of a Collodaria colony section.

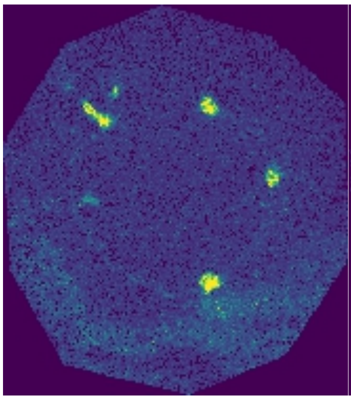

158.0437 ± 0.0050Da + 2.0ppm, Adenine [M+Na]$^+$

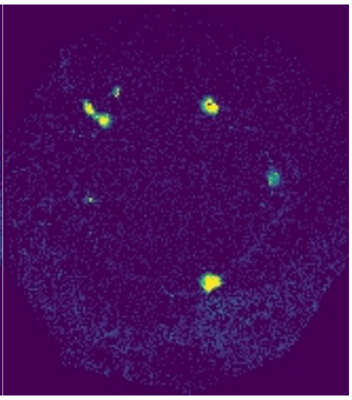

135.0301 ± 0.0050Da + 2.0ppm, Guanine [M-NH_3+H]$^+$

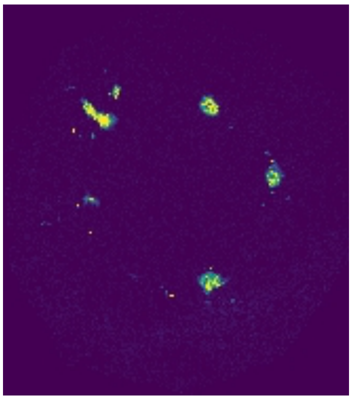

174.0386 ± 0.0050Da + 2.0ppm, Guanine [M+Na]$^+$

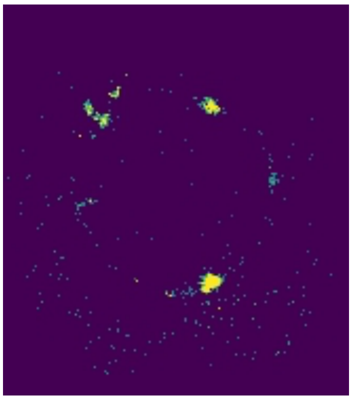

158.0924 ± 0.0050Da + 2.0ppm, Arginine [M-NH_3+H]$^+$

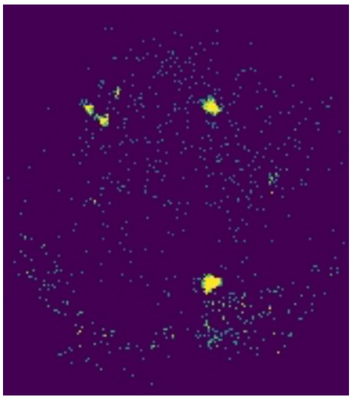

175.1190 ± 0.0050Da + 2.0ppm, Arginine [M+H]$^+$

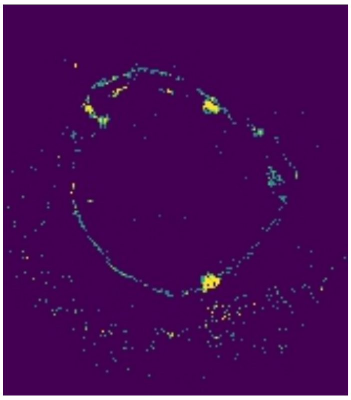

138.0525 ± 0.0050Da + 2.0ppm, Proline [M+Na]$^+$

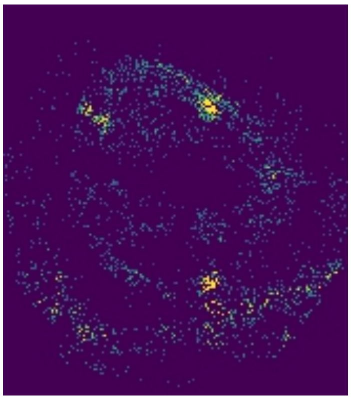

166.0839 ± 0.0050Da + 2.0ppm, Stachydrine [M+Na]$^+$

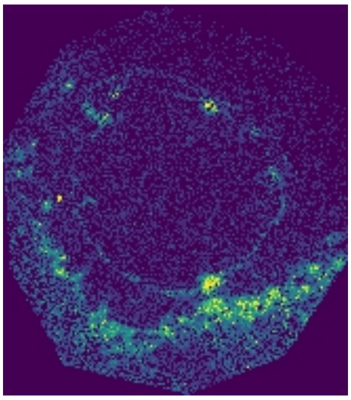

205.0972 ± 0.0050Da + 2.0ppm, Tryptophane [M+H]$^+$

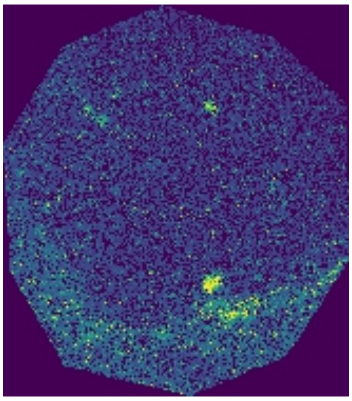

227.0791 ± 0.0050Da + 2.0ppm, Tryptophane [M+Na]$^+$

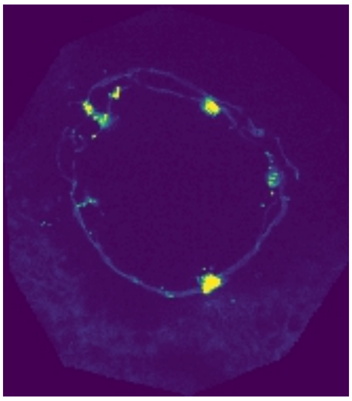

130.0649 ± 0.0050Da + 2.0ppm, [C_9H_8N]$^{\bullet+}$

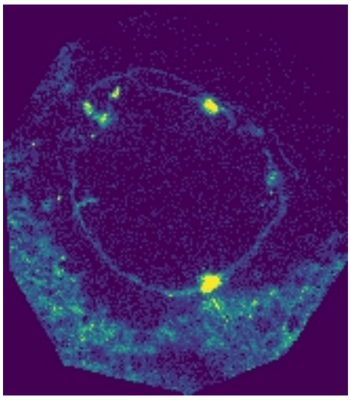

147.0764 ± 0.0050Da + 2.0ppm, Glutamine [M+H]$^+$

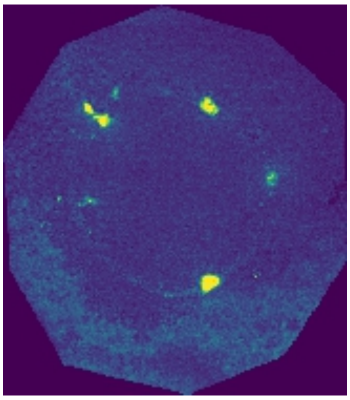

184.0734 ± 0.0050Da + 2.0ppm, γ-Butyrobetaine [M+K]$^+$

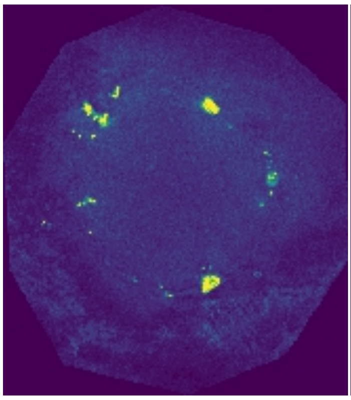

104.1075 ± 0.0050Da + 2.0ppm, Choline [M]$^+$

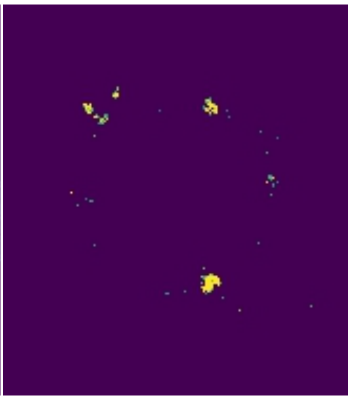

126.0895 ± 0.0050Da + 2.0ppm, Choline [M+Na]$^+$

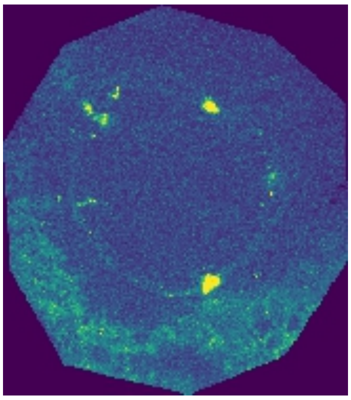

142.0634 ± 0.0050Da + 2.0ppm, Choline [M+K]$^+$

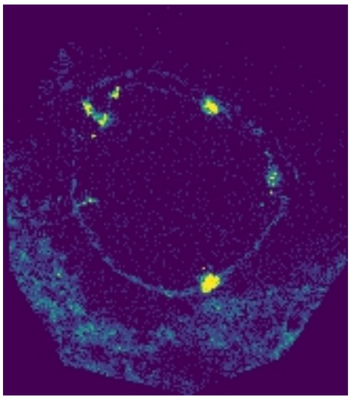

132.0768 ± 0.0050Da + 2.0ppm, Creatine [M+H]$^+$

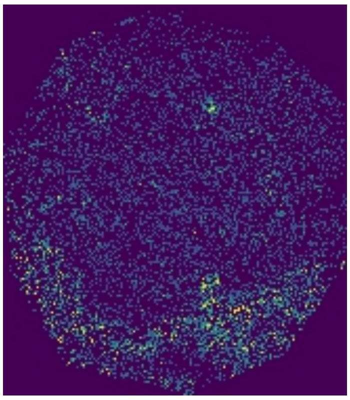

157.0294 ± 0.0050Da + 2.0ppm, DMSP $[M+Na]^+$

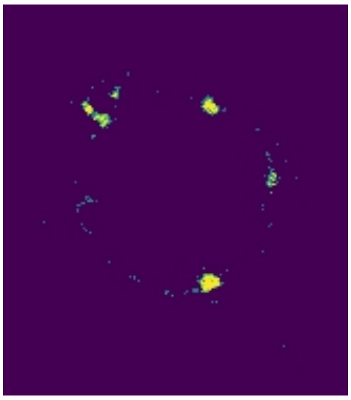

99.0917 ± 0.0050Da + 2.0ppm, Ectoine $[M-CO_2+H]^+$

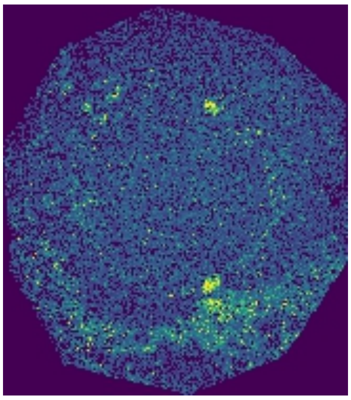

181.0374 ± 0.0050Da + 2.0ppm, Ectoine $[M+K]^+$

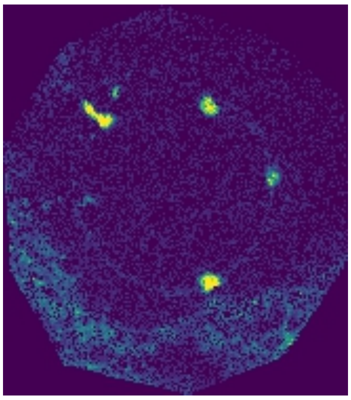

161.0631 ± 0.0050Da + 2.0ppm, Gonyol $[M-H_2O+H]^+$

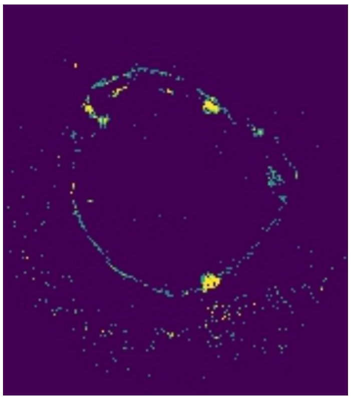

138.0550 ± 0.0050Da + 2.0ppm, Homarine / Trigonelline $[M+H]^+$

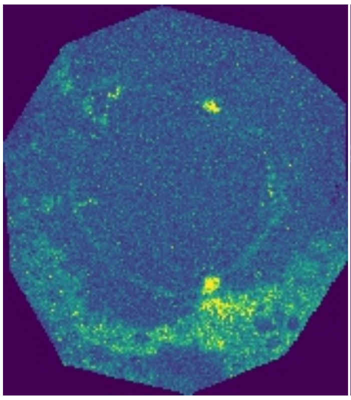

160.0369 ± 0.0050Da + 2.0ppm, Homarine / Trigonelline $[M+Na]^+$

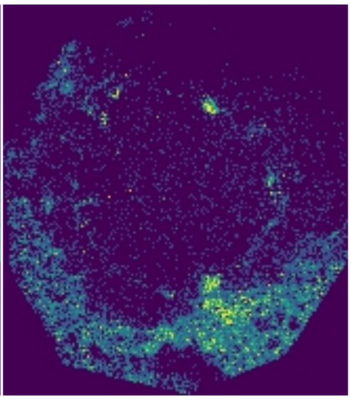

176.0108 ± 0.0050Da + 2.0ppm, Homarine / Trigonelline $[M+K]^+$

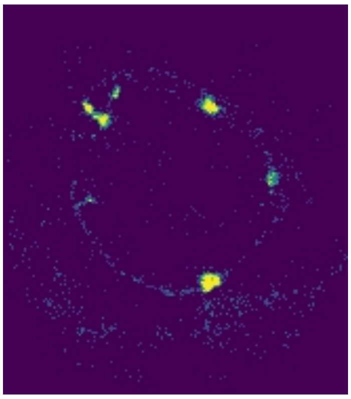

95.0603 ± 0.0050Da + 2.0ppm, Urocanic acid $[M-CO_2+H]^+$

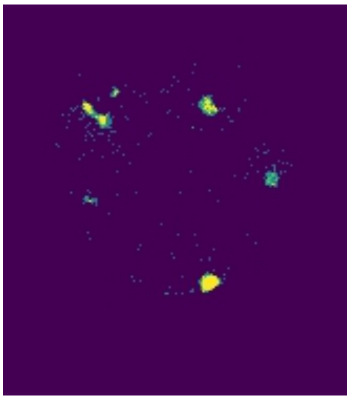

478.3292 ± 0.0050Da + 2.0ppm, 1-Palmitoyl-sn-glycero-3-phosphocholine $M-[H_2O+H]^+$

Appendix S3: Microscopic images

Brightfield microscopy – Image of Collodaria colonies were taken with a stereoscope Leica S AP0 and an INFINITY3-3 camera. Images of Acantharia and the central capsule of Collodaria were taken with a Leica DM IL LED microscope equipped with a Leica DFC280 camera.

Fluorescent microscopy – Fluorescent images of Collodaria colony were taken with SP8 Leica confocal microscope (excitation 638 nm, emission 645–671 nm). Fluorescent image of Acantharia was taken with Viventis LS2 light-sheet microscope (excitation 405 nm, emission 647–800 nm).

Appendix S4: List of standards used for LC-MS measurements.

Ectoine, nicotinate, nicotinamide, GABA betaine hydrochloride, diethanolamine, thiamine, myristoylcarnitine, spermidine, sphingenine from MSMLS, urocanic acid from MSMLS (Sigma Aldrich); betaine, acetylcarnitine hydrochloride, propionylcarnitine, n-dimethylarginine, choline chloride (Sigma); stachydrine (Fluorochem); L-arginine monohydrochloride, L-glutamine, L-leucine, L-phenylalanine, L-proline, L-tyrosine, L-valine, guanine, trigonelline hydrochloride, fucoxanthine, L-isoleucine, L-tryptophane (Fluka analytical); adenosine, pipecolinic acid, hypoxanthine, 1-(2-Hydroxyethyl)piperazine (AlfaAesar); linoleamide (Santa Cruz Biotechnology, Inc.); sulfobetaine (ABCR GmbH); (14:0) 1-myristoyl-sn-glycero-3-phosphocholine, (16:0) 1-palmitoyl-sn-glycero-3-phosphocholine (Avanti Polar Lipids Part of Corda International Plc); adenine (Carl Roth); taurine (Merck KGaA); creatine monohydrate, 3-methyladenine (Thermo Fisher Scientific); phytosphingosine (Tokyo Chemical Industry Co., Ltd); hydroxyproline betaine, β-alanine betaine, alanine betaine (synthetized as described below (Appendix S5); DMSP, homarine, gonyol [1].

Appendix S5: Synthesis of betaines

Since analytical standards for alanine betaine, β-alanine betaine, and hydroxyproline betaine were not available, the betaines were synthesized using a method described in Chen and Benoiton [2] with some modifications. Briefly, 1 g of $KHCO_3$ was dissolved in 20 mL of methanol by stirring for 1 h at 30 °C. Of this solution, 300 μL were added to 3 μg of the corresponding amino acid (alanine, β-alanine, or hydroxyproline). Subsequently, 60 μL of iodomethane were added to each sample, and samples were vortexed for 24 h at room temperature. After that, 100 μL of formic acid were added, samples were dried under vacuum and stored at –20 °C. For LC-HR-MS analysis, samples were dissolved and subsequently diluted 1 : 1000 (*v*:*v*) in either a mixture of methanol: acetonitrile: water (5:9:1, *v*:*v*:*v*) for β-alanine betaine, and hydroxyproline betaine, or in H_2O for alanine betaine. The verification of compounds was done based on their accurate mass.

Appendix S6: LC-MS instrument settings.

LC settings: For both columns column compartment temperature was set to 25°C. Injection volume of Collodaria and *B. nutricula* was 1 µl for both columns, of Acantharia and *P. cordata* samples – 1 µl for HILIC column and 3 µl for C18 column. The auto-injector was not washed between samples, the carryover of the samples was controlled by the solvent blank measurements.

MS settings: MS1 measurements were conducted with the following settings: automatic gain control target – 3E6, the maximum ion injection time – 200 ms, and scan range from 80 to 1200 m/z. Single samples were measured simultaneously with positive and negative modes with resolution 70,000. The order of measurement for samples was randomized.

MS2 measurements were performed for 200 most intense signals among each group of compounds (algal unique compounds, holobiont unique compounds, common compounds) detected with the positive ionization mode. The measurements were performed with positive ionization mode using parallel reaction monitoring with inclusion lists created for each group of compounds. The duration time and runtime were the same as for the MS1 measurement, the settings were as followed: automatic gain control target 2E5, maximum ion injection time 100 ms, three-stepped normalized collision energy 15, 30, 45, scan range from 80 to 1200 m/z, resolution 70,000, in profile mode.

Appendix S7: Sirius settings

Raw files were converted into .mzML format using Proteowizard Suite (proteowizard.sourceforge.net) with vendor peak picking enabled. Putative identification of unknowns was performed depending on the tree fragmentation score [3], and the percentage of CSI:FingerID tool [4]. In case of identification to compound class, the CSI:Finger ID was ≥ 65%, and Posterior probability of the compound class was ≥ 90%.

Appendix S8: List of metabolites identified and confirmed with analytical standards based on measured mass (± 5 ppm), retention time (± 0.2 min), and MS2 spectrum.

Metabolite identification	Chemical formula	Reference Ion	Measured *m/z* for samples, ± 5 ppm	Measured *m/z* standards, ± 5 ppm	Theoretical mass	Mass difference, ppm	Retention time HILIC, ± 0.2 min	Retention time C18, ± 0.2 min
1-myristoy-sn-glycero-3-phosphocholine	C22H46NO7P	$[M+H]^+$	468.3082	468.3083	468.3090	1.71		6.4
1-palmitoy-sn-glycero-3-phosphocholine	C24H50NO7P	$[M+H]^+$	496.3399	496.3396	496.3403	0.81	1.07	8.1
3-methyladenine	C6H7N5	$[M+H]^+$	150.0775	150.0777	150.0779	2.67	1.84	
Acetyl carnitine	C9H17NO4	$[M+H]^+$	204.1234	204.1234	204.1235	0.49		0.66
Adenine	C5H5N5	$[M+H]^+$	136.0621	136.0621	136.0623	1.47	1.05	
Adenosine	C10H13N5O4	$[M+H]^+$	268.1042	268.1039	268.1045	1.12	1	
Alanine betaine	C6H13NO2	$[M+H]^+$	132.102	132.1022	132.1024	3.03	2.3	
Choline	C5H14NO+	$[M+H]^+$	104.1072	104.1071	104.1075	2.88	2.82	
Creatine	C4H9N3O2	$[M+H]^+$	132.0772	132.077	132.0773	0.76	2.89	
Diethanolamine	C4H11NO2	$[M+H]^+$	106.0864	106.0864	106.0868	3.77	2.98	
Dimethylsulfonioacetate	C4H8O2S	$[M+H]^+$	121.0323	121.032	121.0323	0.00	2.3	
Ectoine	C6H10N2O2	$[M+H]^+$	143.0819	143.0819	143.0820	0.70	2.8	0.83
Fucoxanthin	C42H58O6	$[M+H]^+$	659.43	659.4299	659.4311	1.67		8.2
Glycine betaine	C5H11NO2	$[M+H]^+$	118.0866	118.0862	118.0868	1.69	2.35	0.85
Gonyol	C7H14O3S	$[M+H]^+$	179.0739	179.0741	179.0741	1.12	3.52	
Guanine	C5H5N5O	$[M+H]^+$	152.0567	152.0571	152.0572	3.29	1.4	
Homarine	C7H7NO2	$[M+H]^+$	138.0551	138.0551	138.0555	2.90	1.621	
Hydroxyproline betaine	C7H13NO3	$[M+H]^+$	160.0969	160.0969	160.0973	2.50	2.25	
Hypoxanthine	C5H4N4O	$[M+H]^+$	137.0461	137.0463	137.0463	1.46	1.09	0.84
Arginine	C6H14N4O2	$[M+H]^+$	175.1192	175.1193	175.1195	1.71	5.87	
Glutamine	C5H10N2O3	$[M+H]^+$	147.0767	147.0765	147.0769	1.36	3.11	
Linoleamide	C18H33NO	$[M+H]^+$	280.2637	280.2638	280.2640	1.07	0.72	
Isoleucine	C6H13NO2	$[M+H]^+$	132.1022	132.1022	132.1024	1.51	1.55	
Leucine	C6H13NO2	$[M+H]^+$	132.1022	132.1022	132.1024	1.51	1.43	1
Phenylalanine	C9H11NO2	$[M+H]^+$	166.0864	166.0866	166.0868	2.41	1.3	
Proline	C5H9NO2	$[M+H]^+$	116.0708	116.071	116.0711	2.58	2.26	
Proline betaine	C7H13NO2	$[M+H]^+$	144.1021	144.1023	144.1024	2.08	2.41	
Tryptophan	C11H12N2O2	$[M+H]^+$	205.0977	205.0975	205.0977	0.00		2.5
Tyrosine	C9H11NO3	$[M+H]^+$	182.0814	182.0818	182.0817	1.65	1.76	
Valine	C5H11NO2	$[M+H]^+$	118.0864	118.0867	118.0868	3.39	1.88	
Myristoylcarnitine	C21H41NO4	$[M+H]^+$	372.3107	372.311	372.3113	1.61		6
N-(2-Hydroxyethyl) piperazine	C6H14N2O	$[M+H]^+$	131.1183	131.1182	131.1184	0.76	5.747	
Dimethyl arginine	C8H18N4O2	$[M+H]^+$	203.1507	203.1502	203.1508	0.49	5.73	
Nicotinamide	C6H6N2O	$[M+H]^+$	123.0557	123.0558	123.0558	0.81		0.8
Nicotinate	C6H5NO2	$[M+H]^+$	124.0393	124.0393	124.0398	4.03		0.8
Acetycarnitine	C9H17NO4	$[M+H]^+$	204.1233	204.1232	204.1235	0.98	2.3	
Propanoycarnitine	C10H19NO4	$[M+H]^+$	218.1388	218.1388	218.1392	1.83	2.07	
Phytosphingosine	C18H39NO3	$[M+H]^+$	318.3004	318.3003	318.3008	1.26	1.099	
Pipecolic acid	C6H11NO2	$[M+H]^+$	130.0866	130.0868	130.0868	1.54	2.09	
Dimethilsulfonipropionate	C5H10O2S	$[M+H]^+$	135.0477	135.0477	135.0479	1.48	4.16	
Spermidine	C7H19N3	$[M+H]^+$	146.165	146.1655	146.1657	4.79	7.622	

Sphingenine	C18H39NO2	$[M+H]^+$	302.3053	302.3052	302.3059	1.98		6.1
Taurine	C2H7NO3S	$[M+H]^+$	126.022	126.0222	126.0224	3.17	2.5	
Trigonelline	C7H7NO2	$[M+H]^+$	138.0552	138.0552	138.0555	2.17	2.303	
Urocanic acid	C6H6N2O2	$[M+H]^+$	139.0505	139.0506	139.0507	1.44	1.128	
β-Alanine betaine	C6H13NO2	$[M+H]^+$	132.1025	132.1022	132.1024	0.76	3.87	
γ-Butyrobetaine	C7H15NO2	$[M+H]^+$	146.1179	146.1179	146.1181	1.37	2.95	

Appendix S9: List of metabolites identified till compounds class, calculation of molecular formula and identification was performed with Sirius (5.6.3), CSI:Finger ID ≥ 65%, and Posterior probability of the compound class ≥ 90%.

Organisms	Measured *m/z*, ± 5 ppm	Reference Ion	Retention time, min, ± 0.2 min	Formula	Theoretical mass	Mass difference, ppm	Compound class	Column
Acantharia and *P. cordata*	173.0923	$[M+H]^+$	2.4	C7H12N2O3	173.0926	1.7	Dipeptide	HILIC
	246.1813	$[M+H]^+$	6.3	C11H23N3O3	246.1817	1.6	Dipeptide	HILIC
	260.1972	$[M+H]^+$	0.9	C12H25N3O3	260.1974	0.8	Dipeptide	HILIC
	784.4995	$[M+H]^+$	0.7	C45H70NO8P	784.4917	9.9	Phosphatidyl ethanolamine	HILIC
Acantharia only	175.108	$[M+H]^+$	0.9	C7H14N2O3	175.1082	1.1	Dipeptide	C18
	189.1236	$[M+H]^+$	2.0	C8H16N2O3	189.1239	1.6	Dipeptide	C18
	189.1236	$[M+H]^+$	2.0	C8H16N2O3	189.1239	1.6	Dipeptide	C18
	189.1237	$[M+H]^+$	2.0	C8H16N2O3	189.1239	1.1	Dipeptide	C18
	203.1394	$[M+H]^+$	2.2	C9H18N2O3	203.1395	0.5	Dipeptide	C18
	219.134	$[M+H]^+$	2.0	C9H18N2O4	219.1344	1.8	Dipeptide	C18
	223.1079	$[M+H]^+$	2.5	C11H14N2O3	223.1082	1.3	Dipeptide	C18
	233.1496	$[M+H]^+$	2.2	C10H20N2O4	233.1501	2.1	Dipeptide	C18
	237.1234	$[M+H]^+$	2.5	C12H16N2O3	237.1239	2.1	Dipeptide	C18
	247.1289	$[M+H]^+$	1.0	C10H18N2O5	247.1293	1.6	Dipeptide	C18
	260.1606	$[M+H]^+$	1.9	C11H21N3O4	260.161	1.5	Dipeptide	C18
	260.1606	$[M+H]^+$	1.9	C11H21N3O4	260.161	1.5	Dipeptide	C18
	260.197	$[M+H]^+$	0.9	C12H25N3O3	260.1974	1.5	Dipeptide	C18
	261.1446	$[M+H]^+$	2.1	C11H20N2O5	261.145	1.5	Dipeptide	C18
	261.1446	$[M+H]^+$	2.1	C11H20N2O5	261.145	1.5	Dipeptide	C18
	269.1133	$[M+H]^+$	1.5	C12H16N2O5	269.1137	1.5	Dipeptide	C18
	269.1609	$[M+H]^+$	0.9	C12H20N4O3	269.1613	1.5	Dipeptide	C18
	286.151	$[M+H]^+$	2.2	C11H19N5O4	286.1515	1.7	Dipeptide	HILIC
	288.203	$[M+H]^+$	5.9	C12H25N5O3	288.2035	1.7	Dipeptide	C18
	288.2032	$[M+H]^+$	5.9	C12H25N5O3	288.2035	1.0	Dipeptide	HILIC
	294.1449	$[M+H]^+$	2.5	C14H19N3O4	294.1453	1.4	Dipeptide	C18
	294.1812	$[M+H]^+$	1.5	C15H23N3O3	294.1817	1.7	Dipeptide	C18
	296.124	$[M+H]^+$	1.6	C13H17N3O5	296.1246	2.0	Dipeptide	C18
	303.1453	$[M+H]^+$	1.4	C15H18N4O3	303.1457	1.3	Dipeptide	C18
	320.1276	$[M+H]^+$	5.6	C12H21N3O5S	320.128	1.2	Dipeptide	HILIC
	320.1641	$[M+H]^+$	6.2	C13H25N3O4S	320.1644	0.9	Dipeptide	HILIC
	322.1875	$[M+H]^+$	1.8	C15H23N5O3	322.1879	1.2	Dipeptide	C18
	334.1434	$[M+H]^+$	5.3	C13H23N3O5S	334.1436	0.6	Tripeptide	HILIC
	355.1323	$[M+H]^+$	2.2	C16H22N2O5S	355.1327	1.1	Dipeptide	C18
	364.1539	$[M+H]^+$	5.1	C14H25N3O6S	364.1542	0.8	Tripeptide	HILIC
	364.1868	$[M+H]^+$	2.5	C18H25N3O5	364.1872	1.1	Tripeptide	C18

	468.3083	$[M+H]^+$	6.2	C22H46NO7P	468.309	1.5	Lysophosphotidyl choline	C18
	482.3237	$[M+H]^+$	6.8	C23H48NO7P	482.3246	1.9	Lysophosphotidyl choline	C18
	482.324	$[M+H]^+$	6.8	C23H48NO7P	482.3246	1.2	Lysophosphotidyl choline	C18
	500.2769	$[M+H]^+$	6.0	C25H42NO7P	500.2777	1.6	Lysophosphatidyl ethanolamine	C18
	542.3246	$[M+H]^+$	6.3	C28H48NO7P	542.3246	0.0	Lysophosphotidyl choline	C18
	544.3401	$[M+H]^+$	6.8	C28H50NO7P	544.3403	0.4	Lysophosphotidyl choline	C18
	568.3398	$[M+H]^+$	6.7	C30H50NO7P	568.3403	0.9	Lysophosphotidyl choline	C18
	852.5543	$[M+H]^+$	1.3	C50H78NO8P	852.5543	0.0	Phosphatidylcholine	HILIC
P. cordata only	851.5481	$[M+H]^+$	8.2	C46H79N2O10P	851.555	8.1	Phosphatidylcholine	C18
	755.4875	$[M+H]^+$	8.8	C41H71O10P	755.4863	1.6	Phosphatidylglycerol	C18
	755.4879	$[M+H]^+$	8.8	C41H71O10P	755.4863	2.1	Phosphatidylglycerol	C18
	760.499	$[M+H]^+$	9.4	C43H70NO8P	760.4917	9.6	Phosphatidylethanola mine	C18
	772.4915	$[M+H]^+$	9.0	C44H70NO8P	772.4917	0.3	Phosphatidylethanola mine	C18
	772.492	$[M+H]^+$	9.0	C44H70NO8P	772.4917	0.4	Phosphatidylethanola mine	C18
	814.5435	$[M+H]^+$	7.9	C40H80NO13P	814.5445	1.1	Phosphatidylcholine	C18
	816.5247	$[M+H]^+$	7.7	C39H78NO14P	816.5238	1.1	Phosphatidylcholine	C18
	858.5696	$[M+H]^+$	8.5	C42H84NO14P	858.5707	1.3	Phosphatidylcholine	C18
	884.624	$[M+H]^+$	8.5	C52H86NO8P	884.6169	8.0	Phosphatidylcholine	C18
	884.6244	$[M+H]^+$	8.5	C52H86NO8P	884.6169	8.5	Phosphatidylcholine	C18
Collodaria and *B. nutricula*	832.5797	$[M+H]^+$	1.1	C48H82NO8P	832.5856	7.1	Phosphatidylcholine	HILIC
	496.3387	$[M+H]^+$	7.5	C24H50NO7P	496.3403	3.2	Lysophosphatidyl choline	HILIC
	542.3223	$[M+H]^+$	6.3	C28H48NO7P	542.3246	4.2	Lysophosphotidyl choline	C18
	568.3381	$[M+H]^+$	6.7	C30H50NO7P	568.34	3.3	Lysophosphotidyl choline	C18
	626.4238	$[M+H]^+$	7.7	C34H60NO7P	626.4185	8.5	Ether-linked phosphatidyl choline	C18
	640.4392	$[M+H]^+$	7.8	C35H62NO7P	640.4342	7.8	Ether-linked phosphatidyl choline	C18
	744.503	$[M+H]^+$	7.7	C43H70NO7P	744.4968	8.3	Ether-linked phosphatidyl ethanolamine	C18
	878.5667	$[M+H]^+$	1.3	C52H80NO8P	878.5699	3.6	Phosphatidylcholine	HILIC
Collodaria only	600.4081	$[M+H]^+$	7.6	C32H58NO7P	600.4029	8.7	Lysophosphatidyl choline	C18
	604.4034	$[M+H]^+$	7.0	C31H58NO8P	604.3978	9.3	Phosphatidylcholine	C18
B. nutricula only	746.5183	$[M+H]^+$	7.4	C40H77NO7P2	746.5253	9.4	Phosphatidylcholine	C18
	452.2743	$[M+H]^+$	6.2	C21H42NO7P	452.2777	7.5	Lysophosphatidyl ethanolamine	C18
	232.1401	$[M+H]^+$	6.2	C8H17N5O3	232.1409	3.4	Dipeptide	HILIC
	246.1807	$[M+H]^+$	6.3	C11H23N3O3	246.1817	4.1	Dipeptide	HILIC
	247.1287	$[M+H]^+$	1.0	C10H18N2O5	247.1293	2.4	Dipeptide	C18
	303.1771	$[M+H]^+$	6.1	C11H22N6O4	303.178	3.0	Dipeptide	HILIC
	350.1561	$[M+H]^+$	2.3	C13H23N3O8	350.1563	0.6	Tripeptide	C18
	376.1705	$[M+H]^+$	2.2	C15H25N3O8	376.1719	3.7	Tripeptide	C18
	408.1427	$[M+H]^+$	2.3	C15H25N3O8S	408.144	3.2	Tripeptide	C18
	468.3076	$[M+H]^+$	6.2	C22H46NO7P	468.309	3.0	Lysophosphotidyl choline	C18
	480.3073	$[M+H]^+$	6.9	C23H46NO7P	480.309	3.5	Lysophosphatidyl ethanolamine	C18
	480.3073	$[M+H]^+$	6.9	C23H46NO7P	480.309	3.5	Lysophosphatidyl ethanolamine	C18

	496.3385	$[M+H]^+$	7.5	C24H50NO7P	496.3403	3.6	Lysophosphatidyl choline	C18
	514.2908	$[M+H]^+$	5.8	C26H44NO7P	514.2933	4.9	Lysophosphatidyl choline	C18
	516.3075	$[M+H]^+$	6.0	C26H46NO7P	516.309	2.9	Lysophosphatidyl choline	C18
	554.3244	$[M+H]^+$	6.4	C29H48NO7P	554.3246	0.4	Lysophosphatidyl choline	C18
	608.3906	$[M+H]^+$	7.0	C30H58NO9P	608.3927	3.5	Phosphatidyl ethanolamine	C18
	636.4219	$[M+H]^+$	8.1	C32H62NO9P	636.424	3.3	Phosphatidyl ethanolamine	C18
	692.383	$[M+NH4]^+$	5.6	C33H54O14	692.3857	3.9	Glycerolipids	C18
	764.5077	$[M+H]^+$	9.2	C39H74NO11P	764.5077	0.0	Phosphatidylcholine	C18
	766.5234	$[M+H]^+$	10.5	C39H76NO11P	766.5234	0.0	Phosphatidylcholine	C18
	766.5234	$[M+H]^+$	10.2	C39H76NO11P	766.5234	0.0	Phosphatidylcholine	C18
	772.5334	$[M+H]^+$	8.4	C45H74NO7P	772.5281	6.9	Lysophosphatidyl ethanolamine	C18
	774.5496	$[M+H]^+$	8.1	C45H76NO7P	774.5437	7.6	Phosphatidylcholine	C18

Appendix S10: MALDI / MALDI-2 profiling of analytical standards

The MALDI-matrices CHCA, DHAP, and DHB (all Bruker, Bremen, Germany) were tested against a panel of 32 compounds, previously identified in the metabolomic experiments. To that purpose, 2 µL of each liquid standard (Appendix S4) were spotted three times on a ground steel MALDI target (Bruker) and dried in a desiccator shuttle. Each cluster of spotted standards was then sprayed with one of the three matrices in a M3+ sprayer (HTXImaging, Chapel Hill NC, USA), using the methods described below (Appendix S9). Mass spectra were acquired in positive ion mode on a timsTOF fleX MALDI-2 mass spectrometer (Bruker) with and without laser post-ionization, after calibrating and tuning the system for optimal detection with red phosphorous (Appendix S12, S13). The data was analysed in DataAnalysis 6.1 (Bruker) and subsequently visualized in R, Version 4.4.1 [5], using the ggplot2 package [6].

Appendix S11: Matrix application methods used for applying MALDI matrices to dried liquid standards on a MALDI target plate and cryosections with the M3+ sprayer.

Matrix	CHCA	DHB	DHAP
Solvent	3:1 ACN/H2O + 0.1% FA	3:1 MeOH/H2O + 0.1% FA	3:1 ACN/H2O + 0.1% FA
Concentration in [mg/mL]	10	50	20
Nozzle temp. [°C]	80	90	80
Nebulizer gas pressure [psi]	10	10	10
FLOW RATE [µl/min]	100	75	50
Nozzle velocity [mm/min]	1250	1200	1200
Track spacing [mm]	2	2	2
Number of layers	5	5	5
Nozzle meandering	horizontal and vertical	horizontal and vertical	horizontal and vertical
Drying time [s]	30	15	30

Appendix S12: Measured ion intensities of dried liquid standards acquired with MALDI and MALDI-2 and three different matrices – CHCA, DHAP, and DHB; Each value is based on the integration of 1000 singular spectra, acquired by randomly walking in 2 mm radius every 10 shots and a laser energy (E) set to 50% and 15%, in the MALDI and MALDI-2 experiment, respectively. The liquid standards were spotted and dried prior to the matrix application.

Compound	Reference ion	Exact mass [Da]/ Observed *m/z*	MALDI E=50%			MALDI-2 E=15%		
			DHAP	DHB	CHCA	DHAP	DHB	CHCA
Choline	$[M]^+$	104.1070/104.1071	4954931	206788	4998204	16132439	5438562	21774266
Proline	$[M+H]^+$	116.0706/116.0706	124674	421	181	284214	20288	48421
Valine	$[M+H]^+$	118.0863/118.0864	1011	419	3483	62575	8786	17031
Betaine	$[M+H]^+$	118.0863/118.0863	16780	0	7262	3021204	471708	3741286
Sulfobetaine	$[M+H]^+$	121.0318/121.0317	58138	13720	1510	723903	43026	165267
Taurine	$[M+H]^+$	126.0219/126.0219	2469	203	15638	5388	1163	116605
Pipecolate	$[M+H]^+$	130.0863/130.0864	145809	11490	66	1469786	174930	80955
Creatine	$[M+H]^+$	132.0768/132.0769	707055	67819	756299	511408	94601	265075
Isoleucine	$[M+H]^+$	132.1019/132.1018	2913	270	551	16298	4916	42721
Leucine	$[M+H]^+$	132.1019/132.1017	2798	100	114	47665	4473	187295
Alanine betaine	$[M+H]^+$	132.1019/132.1017	32568	5877	42316	238621	34429	180726
β-alanine betaine	$[M+H]^+$	132.1019/132.1018	234386	65736	979	434678	137734	1394389
DMSP	$[M+H]^+$	135.0474/135.0475	1002478	34344	2151095	2104806	254106	2384499
Adenine	$[M+H]^+$	136.0618/136.0619	1691795	100017	2023584	7433603	999917	6942593
Homarine	$[M+H]^+$	138.0550/138.0554	70799	19135	192708	613869	97916	500666
Trigonelline	$[M+H]^+$	138.0550/138.0554	1444975	379965	1831846	6285249	866113	3713875
Ectoine	$[M+H]^+$	143.0815/143.0819	5242879	159715	2299955	15570762	1338777	9005446
Stachydrine	$[M+H]^+$	144.1019/144.1019	3404507	28297	6044	15072645	1012819	8401989
γ-butyrobetaine	$[M+H]^+$	146.1176/146.1180	475383	62997	14575	1133828	210193	2095191
Glutamine	$[M+H]^+$	147.0764/147.0766	2779	493	6925	93600	14581	164229
Guanine	$[M+H]^+$	152.0567/152.0566	31751	1072	32650	432217	14734	236882
Phenylalanine	$[M+H]^+$	166.0862/166.0868	6420	530	13817	446214	86272	970758
Arginine	$[M+H]^+$	175.1190/175.1193	240405	226463	2447430	3343689	1256281	6930484
Gonyol	$[M+H]^+$	179.0736/179.0738	11942	7286	24933	29450	15912	28853
Tyrosine	$[M+H]^+$	182.0812/182.0810	17032	743	3627	342991	158949	994382
N-dimethylarginine	$[M+H]^+$	203.1503/203.1506	3675647	191024	2436833	11912504	1592001	16045597
Tryptophane	$[M+H]^+$	205.0972/205.0972	6324	337	831	2075917	606857	3874878
Propionylcarnitine	$[M+H]^+$	218.1387/218.1387	2072645	93536	3060409	5024218	522685	5912747
Adenosine	$[M+H]^+$	268.1040/268.1041	1057315	123643	52962	731699	420388	227070
Sphingenine	$[M+H]^+$	302.3054/302.3052	659	686	320	905	2074	917
1-Myristoyl-sn-glycero-3-phosphocholine	$[M+H]^+$	468.3085/468.3080	545799	212106	892198	6723173	992205	3266230
1-Palmitoyl-sn-glycero-3-phosphocholine	$[M+H]^+$	496.3398/496.3396	1693255	139925	1521573	5338927	1642067	4785284
Sum Intensities			28978321	2155157	24840918	107658445	18539463	104496607
Average Intensity			905573	67349	776279	3364326	579358	3265519
Median intensity			135242	16428	20286	727801	166940	982570

Appendix S13: Bar graph of measured intensities ionizing a panel of analytical standards with MALDI and MALDI-2 and three different matrices CHCA, DHAP, and DHB; The liquid standards were spotted and dried prior to the matrix application.

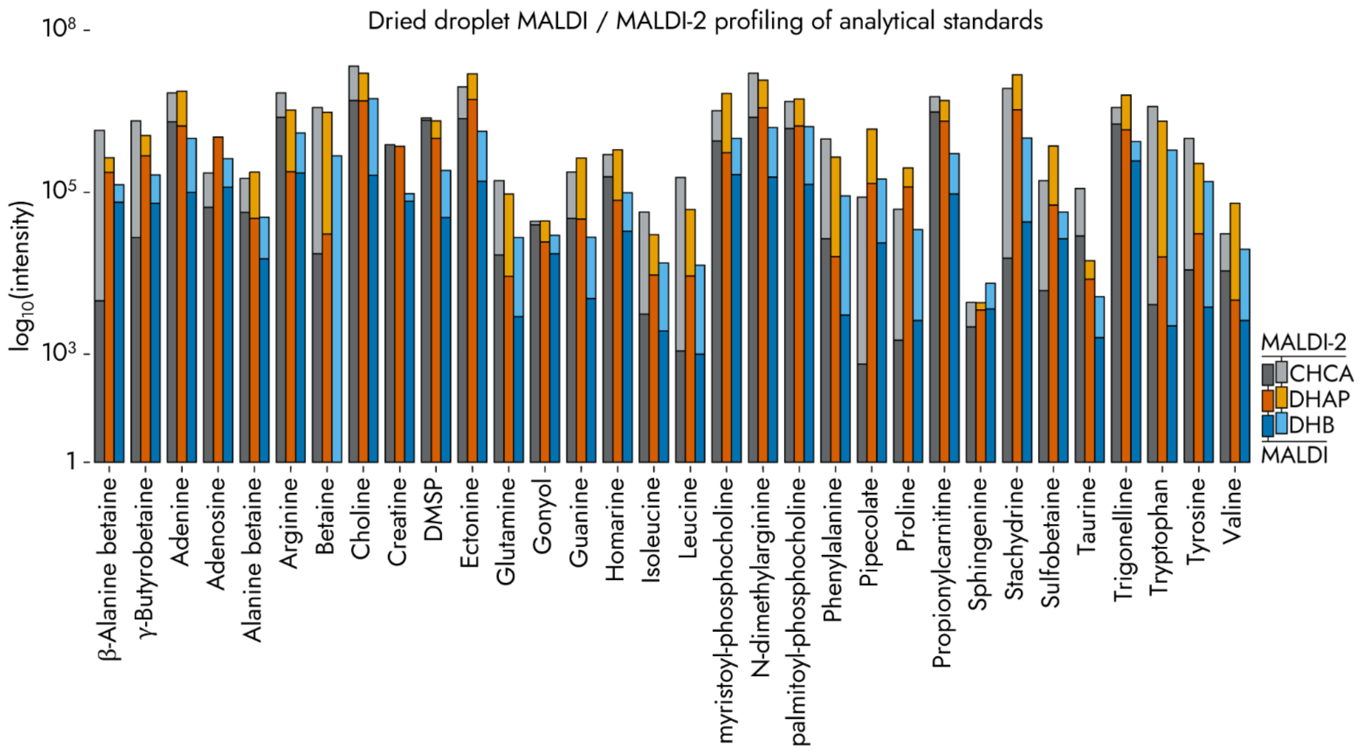

Appendix S14: Possible origin of *N*-(2-hydroxyethyl)piperazine in algal samples.

N-(2-hydroxyethyl)piperazine was detected for both cultures of the free-living algae but not in the radiolarian samples. However, since no information about the bioactive role of this molecule could be found in the literature, it was assumed, that the origin of the compound could be the medium that contained 4-(2-hydroxyethyl)-1-piperazineethanesulfonic acid (HEPES) as a buffer. The higher content in the algal samples could be explained by the adsorption of the compounds on the surface of the algae.

References

1. Gebser B, Pohnert G. Synchronized Regulation of Different Zwitterionic Metabolites in the Osmoadaption of Phytoplankton. *Mar Drugs* 2013; **11**: 2168–2182.

2. Chen FCM, Benoiton NL. A new method of quaternizing amines and its use in amino acid and peptide chemistry. *Can J Chem* 1976; **54**: 3310–3311.

3. Böcker S, Dührkop K. Fragmentation trees reloaded. *J Cheminform* 2016; **8**: 1–26.

4. Dührkop K, Shen H, Meusel M, Rousu J, Böcker S. Searching molecular structure databases with tandem mass spectra using CSI : FingerID. *Proc Natl Acad Sci* 2015; **112**: 12580–12585.

5. Team RC. R: A language and environment for statistical computing. 2021. R Foundation for

Statistical Computing, Vienna, Austria.

6. Wickham H. ggplot2: Elegant graphics for data analysis, 2nd ed. 2016. Springer.
